# Bilateral Inferior Rectus Schwannoma and Strabismus: A Case Report

**DOI:** 10.7759/cureus.55582

**Published:** 2024-03-05

**Authors:** Estefania Ramirez Marquez, Alejandra Santiago, Angel G Torres, Yannina Colon, Roman Velez, Luis Serrano, Joseph Campbell, Ricardo Rodriguez Rosa

**Affiliations:** 1 Ophthalmology, University of Puerto Rico, Medical Sciences Campus, San Juan, USA; 2 Pathology and Laboratory Medicine, University of Puerto Rico, Medical Sciences Campus, San Juan, USA; 3 Neuro-Ophthalmology, University of Puerto Rico, Medical Sciences Campus, San Juan, USA; 4 Pediatric Ophthalmology, University of Puerto Rico, Medical Sciences Campus, San Juan, USA

**Keywords:** pediatrics, strabismus, neurofibromatosis type 2, ocular disease, schwannoma

## Abstract

Schwannomas, also known as neurilemomas, are peripheral nerve sheath neoplasms. They can be sporadic or associated with genetic syndromes including neurofibromatosis type 2 (NF2). Schwannomas may lead to symptoms by exerting pressure on nearby structures, such as nerve and muscle fibers. In this study, we present the case of a 22-year-old female with a history of NF2 who, upon examination, presented with a visibly enlarged salmon-colored mass involving the left inferior rectus that she had since the age of 12 years. Ocular examinations revealed a small left hypertropia and exotropia in all gazes. Magnetic resonance imaging confirmed bilateral involvement of the inferior rectus muscles. She had a partial excisional biopsy of the mass involving the left inferior rectus muscle that confirmed the presence of schwannoma. This case highlights the importance of comprehensive evaluation of sensory and motor functions as well as considering orbital schwannomas in cases of strabismus, especially within the context of neurofibromatosis.

## Introduction

Schwannomas, also termed neurilemmoma, are neoplasms originating from the sheath of peripheral nerves [1-4]. These tumors are histologically characterized by the presence of a fibrous capsule containing the parent nerve, nuclear palisading, and Antoni A and B patterns [1,4,5]. Most schwannomas occur sporadically; nevertheless, they may be associated with genetic syndromes, such as neurofibromatosis type 2 (NF2) [2,3,5]. There are no specific predilections as to race or sex, and the majority of patients present from the age of 20 to 50 years [2,3,6].

Schwannomas account for less than 2% of all orbital tumors [5,7]. Schwannomas are typically asymptomatic; however, proptosis and diplopia are the most common symptoms of orbital schwannomas [1,3,5]. They may also produce a cosmetic deformity or deficits in optic nerve function, depending on the size and location of the masses [4]. Schwannomas tend to be benign neoplasms encapsulated and generally do not infiltrate surrounding structures; therefore, allowing for total surgical removal in most cases, with recurrence being rare [1,5]. We herein present a case of a 22-year-old female with a schwannoma involving her bilateral inferior rectus muscle. The presence of the schwannoma led to inferior rectus muscle atrophy, which in turn resulted in strabismus.

## Case presentation

A 22-year-old female presented to the clinic with a 10-year history of an enlarging mass and swelling in her left lower eyelid. She denied any associated pain, diplopia, loss of vision, or any other ocular complaint. The patient had a past medical history including mental retardation, neurofibromatosis type II, cardiac arrhythmia, bilateral CN VII schwannoma for which she had received radiation therapy, and left CN VII facial palsy. Her cardiac arrhythmia was stable with the use of metoprolol. Her ocular history was remarkable for corneal scars and an isolated retinal hamartoma in the left eye. The patient had delayed seeking treatment as the patient’s mother had not seen the mass increase in size until six months before. Her review of systems and family histories was otherwise unremarkable.

**Figure 1:**
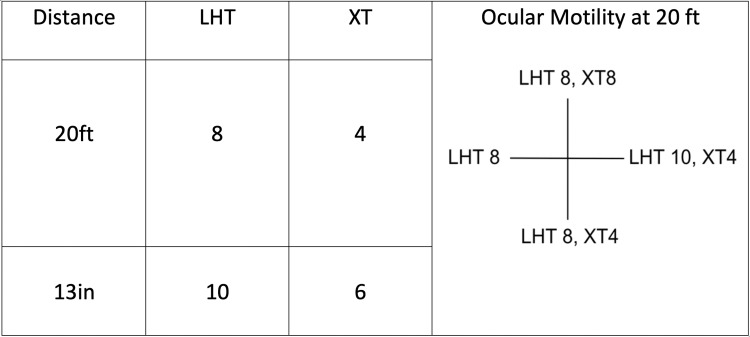
Ocular motility: a small left hypertropia and exotropia were present at distance and near in all gaze positions associated with full ductions and versions. LHT: left hypotropia; XT: exotropia

Upon a comprehensive ophthalmic evaluation, her best-corrected visual acuity was 20/25 in the right eye and 20/100 in the left eye. The pupils were round and reactive to light, and there was no afferent pupillary defect. The manifest refraction for the right eye was -1.75 diopters with a cylindrical correction of -1.25 diopters at an axis of 180 degrees. For the left eye, the manifest refraction was -2.00 diopters with a cylindrical correction of -1.00 diopters at an axis of 180 degrees. Her intraocular pressure was 13 mmHg in both eyes. An Ishihara color-plate test was used to assess the patient’s color vision and revealed no defect in either eye. A sensory-motor test revealed no stereopsis, and the ductions and versions were full. There was no evidence of torsion on the double Maddox rod test. There was suppression of the left eye at 20 feet and 13 inches on the Worth four dot test. The ocular motility test revealed a small left hypertropia and exotropia in all gaze positions (Figure 1).

**Figure 2:**
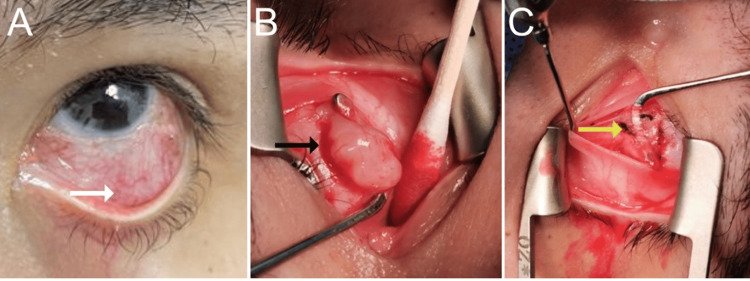
Pre-operative, intra-operative, and status post-partial extraction external view The images show (A) pre-operative external view of the salmon-colored mass (white arrow) extending from the inferior fornix anteriorly, (B) intra-operative external view of the mass (black arrow) seen involving the inferior rectus muscle, and (C) an external view status post-partial extraction of the mass reveals the inferior rectus muscle insertion (yellow arrow) appears atrophic.

The Hertel exophthalmometric values (base 93) were 16 in the right eye and 15 in the left eye, with no evidence of proptosis. On visual field confrontation testing, no field loss was detected in either eye. Humphrey visual field test was performed; however, the results were unreliable as the patient had a history of mental retardation and could not successfully complete the test. External examination was remarkable for lagophthalmos and a salmon-colored mass extending from the inferior fornix anteriorly of the left eye (Figures 2A-2C). Slit-lamp examination was normal for the right eye and showed a central corneal scar on the left eye. The dilated fundus examination was remarkable for a retinal hamartoma on her left eye; the rest of the examination was otherwise normal for both eyes.

**Figure 3:**
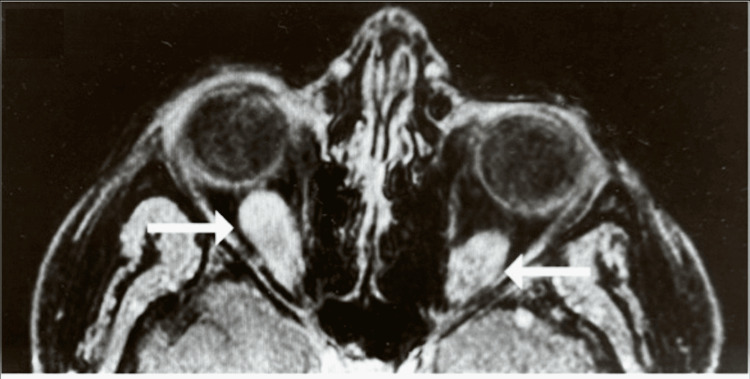
Bilateral enlargement of inferior rectus muscles (arrows) T1 axial view.

**Figure 4:**
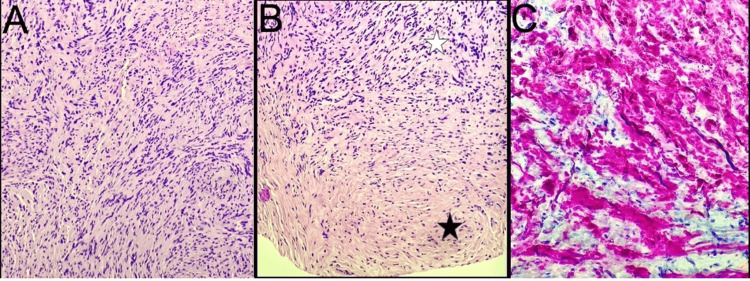
Histopathology of the left eye inferior rectus muscle mass. The images show (A) spindle cell tumor composed of (hematoxylin and eosin stain 200×) (B) compact hypercellular areas (Antoni A tissue; white star), loose microcystic areas (Antoni B tissue; black star), and (C) a strong diffuse pattern (S100 immunohistochemistry; 100×).

The magnetic resonance imaging revealed bilateral enlargement of the inferior rectus muscles, which was unchanged from previous images (Figure 3). A surgical biopsy and excision of the left-sided orbital mass were performed. The salmon-colored mass was found tethered to the inferior rectus muscle and was subsequently dissected. It measured 1.1 cm vertically and 0.5 cm horizontally (Figures 2A-2C). The biopsy result was remarkable for a peripheral nerve sheath tumor, also called schwannoma (Figures 4A-4C).

The patient was seen for subsequent post-operative care during which she denied diplopia, loss of vision, or any ocular complaint. Upon examination, her left eye visual acuity remained stable, and she had complete extraocular movements. Slit lamp examination remained unchanged. One year after the surgery, she had no new complaints, and her examination remained stable. She was later scheduled for continued monitoring of her right orbital mass as well as the recurrence of the left orbital mass.

## Discussion

Neurofibromatosis is a group of rare genetic syndromes characterized by the formation of tumors of the central and peripheral nervous systems [2,3,5]. The patient presented in this case had a known history of NF2 associated with bilateral vestibular schwannomas. The most common ocular complication linked to NF2 is subcapsular cataract, which appears in 80% of NF2 patients [3]. Orbital schwannomas have been reported in as few as 1% neurofibromatosis patients [1,4,6]. The size and precise anatomical location of a particular orbital schwannoma may lead to unique ocular symptoms.

Patients with orbital schwannomas may present a variety of symptoms, including proptosis, orbital muscle atrophy, hypoglobus, cranial nerve dysfunction, and diplopia [1,5-7]. Orbital schwannomas can cause extraocular muscle atrophy and dysfunction, which is the rationale for these tumors resulting in strabismus. These lesions may directly infiltrate ocular muscles or compress those adjacent nerves that are involved in ocular motility, including CN III, CN IV, and CN VI. To the best of our knowledge, this patient represents the first documented case of orbital schwannomas involving the bilateral inferior rectus muscles. Her left inferior rectus muscle was found to be atrophied, most likely due to direct compression by the schwannoma, which reduced the muscle’s ability to contract and relax. Consequently, the patient's small left hypertropia may be explained by the atrophic changes found at the left rectus muscle insertion. Interestingly, the schwannoma involving the right inferior rectus muscle was readily distinguishable on magnetic resonance imaging; however, there was no clinical sign or indication of its presence during the external and slit-lamp examinations.

The primary complaint of patients with orbital schwannomas is proptosis [1,4-7]. However, the patient described in this case went to the clinic for further evaluation of an enlarging mass on the left inferior rectus muscle, but without any additional ocular complaint. The meticulous evaluation of sensory and motor functions is crucial for both the detection of strabismus and the assessment of the fusional status of the patient. In this case, the patient had a small hypertropia and exotropia left eye (OS). Furthermore, evaluating a given patient’s visual acuity, refractive status, and color vision is also vital for monitoring the impact of that patient’s tumor on the optic nerve.

The identification and treatment of orbital schwannoma may ensure that patients are managed appropriately, improve visual outcomes, and prevent long-term ocular morbidity. It is possible that some cases of schwannomas will be incorrectly labeled as idiopathic because of a lack of clinical suspicion; however, increasing awareness about the potential presence of a schwannoma may help ensure timely and precise management. In conclusion, this case highlights the significance of including schwannomas in the differential diagnosis of patients with NF2 and an orbital mass involving extraocular muscles.

## Conclusions

The present case emphasizes the significance of considering orbital schwannomas in the differential diagnosis of strabismus, particularly within the context of neurofibromatosis. It also highlights the potential for the presence of bilateral schwannoma with orbital muscle involvement, even in instances in which the patient presents unilateral ocular signs or symptoms. This is the first documented instance of orbital schwannomas involving the bilateral inferior rectus muscles. Thorough assessments of sensory and motor functions are essential in guiding the appropriate management of this disease.
